# Case Report: ^68^Ga-FAPI PET/CT, a more advantageous detection mean of gastric, peritoneal, and ovarian metastases from breast cancer

**DOI:** 10.3389/fonc.2022.1013066

**Published:** 2022-10-26

**Authors:** Tianyue Li, Xiaojing Jiang, Zhaoqi Zhang, Xiaolin Chen, Jianfang Wang, Xinming Zhao, Jingmian Zhang

**Affiliations:** ^1^ Department of Nuclear Medicine, The Fourth Hospital of Hebei Medical University, Shijiazhuang, China; ^2^ Hebei Provincial Key Laboratory of Tumor Microenvironment and Drug Resistance, Shijiazhuang, China

**Keywords:** ^18^F-FDG, ^68^Ga-FAPI, PET/CT, breast cancer, gastric metastasis, peritoneal carcinomatosis, ovarian metastasis

## Abstract

Breast cancer is the most common malignant tumor in adult women. Its common metastatic sites are lymph nodes, bones, lungs, the liver, and the brain. It is so rare for a patient with breast cancer to have metastases of the gastrointestinal tract, peritoneum, and ovary at the same time that the clinical reporting rate is low. We present a case of a 61-year-old woman who underwent right mastectomy and chemoradiotherapy 3 years ago because of mixed invasive ductal-lobular breast cancer. This time, she came to the hospital due to the symptom of stomach discomfort for 2 weeks. The gastroscopy biopsy result showed gastric metastasis from breast cancer. Then, ^18^F-FDG imaging and ^68^Ga-FAPI PET/CT imaging were performed for further diagnosis; ^68^Ga-FAPI PET/CT demonstrated a significantly elevated FAPI activity in the thickened gastric wall, peritoneum, and bilateral adnexal areas, which was superior to that of ^18^F-FDG. Finally, a biopsy of suspicious lesions was taken for pathological and histochemical examination, which confirmed that, in addition to the gastric metastasis, the peritoneum and bilateral ovaries were all consistent with metastatic breast cancer.

## Case presentation

A 61-year-old woman presented at a gastrointestinal surgery clinic with the symptom of stomach discomfort for 2 weeks. She underwent right modified radical mastectomy and axillary lymph node dissection for invasive ductal-lobular breast cancer (stage pT1cN1M0) 3 years ago, followed by chemotherapy (docetaxel 120 mg, doxorubicin 40 mg, and cyclophosphamide 80 mg every 21 days for a total of six cycles) and 5 weeks of radiotherapy (a total dose of 50 Gy in 25 fractions). Moreover, she has been taking anastrozole up to now. Meanwhile, she also brought her local gastroscopic biopsy result, which showed gastric metastasis from breast cancer. The patient underwent subsequent auxiliary examinations to assess her general condition. Laboratory examination revealed increased levels of CA125 and CA153, which were 46.46 U/ml (reference range 0–25.0) and 158.20 U/ml (reference range 0–24.0), respectively. Contrast-enhanced computed tomography (CECT) demonstrated that the thickening muscularis and mucosa at the distal end of the antrum had moderate enhancement. Pelvic ultrasound demonstrated abnormal changes in the bilateral adnexal areas, but the nature of these changes was uncertain. The patient was then referred to the nuclear medicine department for positron emission tomography/computed tomography (PET/CT) imaging to further evaluate whether there was metastasis in other parts of her body.

**Figure 1:**
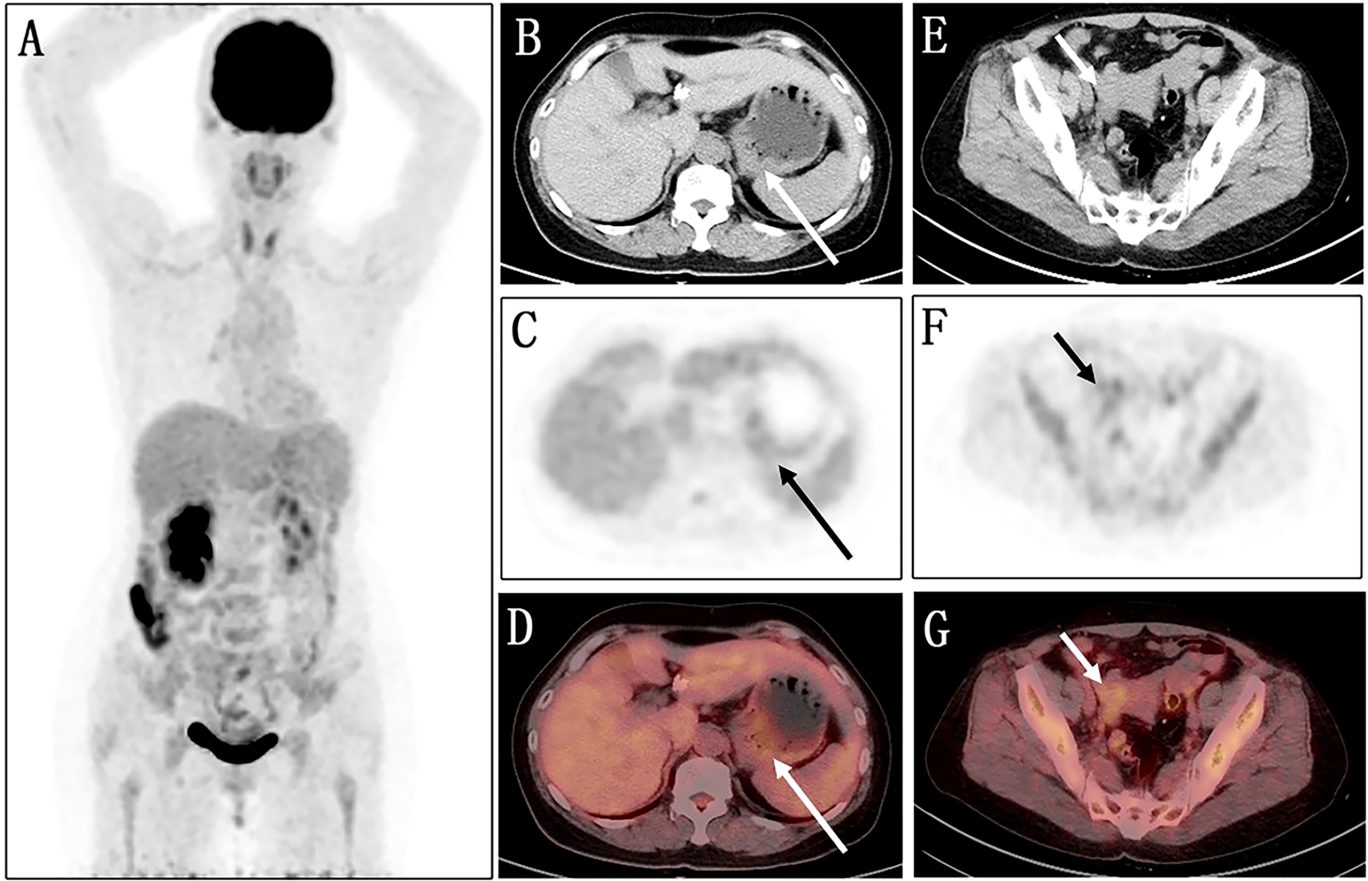
Images of ^18^F-FDG PET/CT. **(A)** No obvious abnormality is found in the maximum intensity projection (MIP) image. **(B–D)** The axial images of the abdominal cavity reveal slight FDG activity in the thickened gastric wall (long arrows; SUV_max_ 3.8). **(E–G)** Similarly, the axial images of the pelvic cavity show an abnormal mass with a slight FDG uptake in the right adnexal area (short arrows; SUV_max_ 3.0).

**Figure 2:**
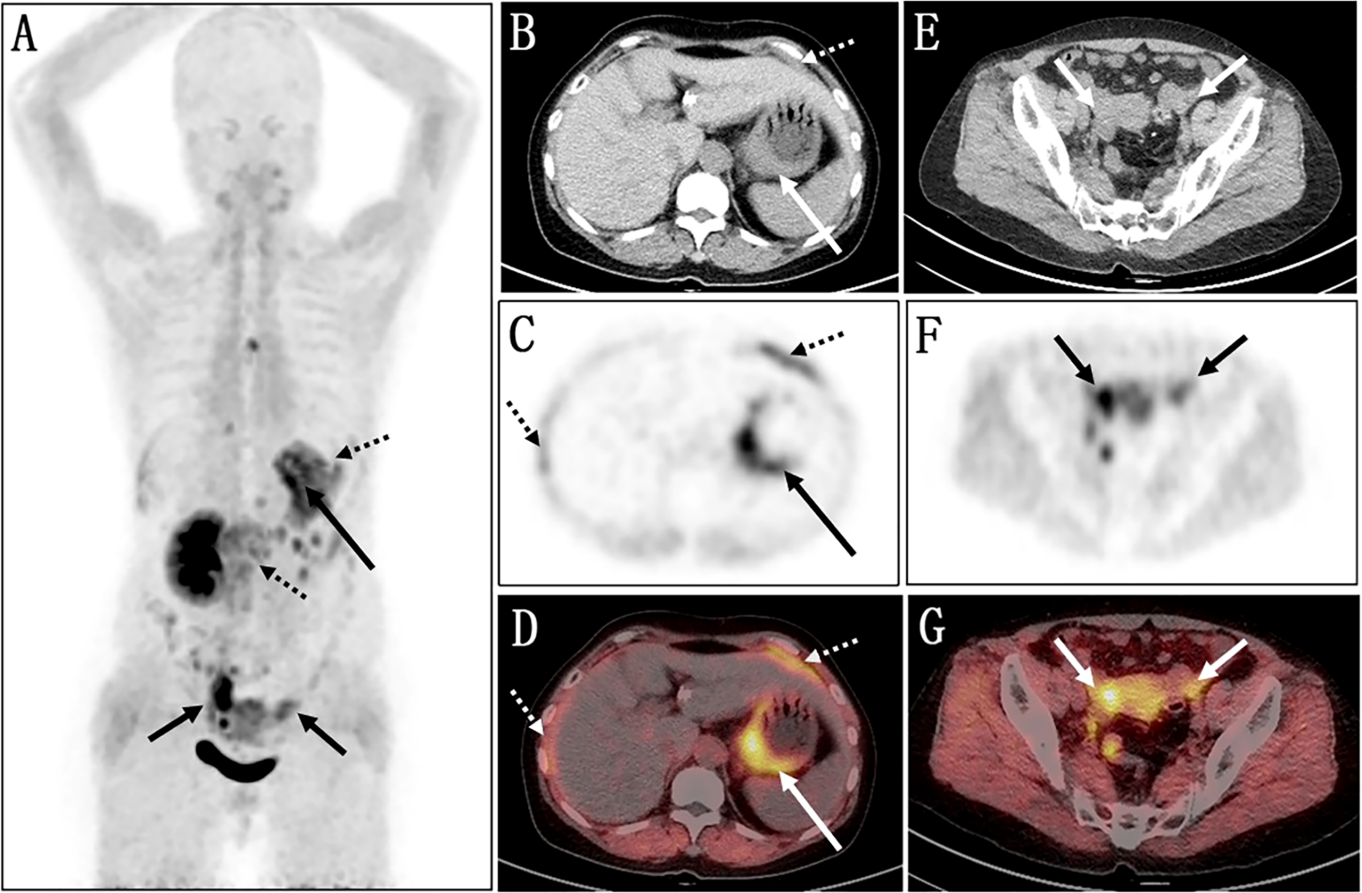
Images of ^68^Ga-FAPI PET/CT. **(A)** The MIP image shows several lesions with varying degrees of FAPI uptake. **(B–D)** Axial views of abdominal cavity demonstrate significantly elevated FAPI activity in the thickened gastric wall (long arrows; SUV_max_ 9.8). **(E–G)** The axial images of pelvic cavity reveal abnormal FAPI uptake in the bilateral adnexal areas (short arrows; SUV_max_ of right 11.6, SUV_max_ of left 6.5). **(C, D)** Furthermore, ^68^Ga-FAPI PET/CT showed extensive peritoneal carcinomatosis with FAPI uptake (dotted arrows; SUV_max_ 3.9).

Fluorine 18 (18F) fluorodeoxyglucose (FDG) PET/CT (**Figure 1**) results revealed slight FDG activity in the thickened gastric wall (SUVmax 3.8) and an abnormal mass in the right adnexal area (SUVmax 3.0). There was no obvious abnormal FDG uptake in the peritoneum or the left adnexal area. In order to assist in making a diagnosis, the patient was recruited in the gallium 68 (68Ga)-conjugated fibroblast-activation protein inhibitor (FAPI) PET/CT clinical trial of malignant tumor approved by the institutional review committee of our hospital after the patient’s written informed consent was obtained (No. 2021069). Compared with ^18^F-FDG, ^68^Ga-FAPI PET/CT (**Figure 2**) results demonstrated significantly elevated FAPI activity in the thickened gastric wall (SUVmax 9.8) and a mass in the right adnexal area (SUVmax 11.6). Furthermore, ^68^Ga-FAPI PET/CT showed extensive peritoneal carcinomatosis with FAPI uptake (SUVmax 3.9) and a mass in the left adnexal area with moderate FAPI activity (SUVmax 6.5).

**Figure 3:**
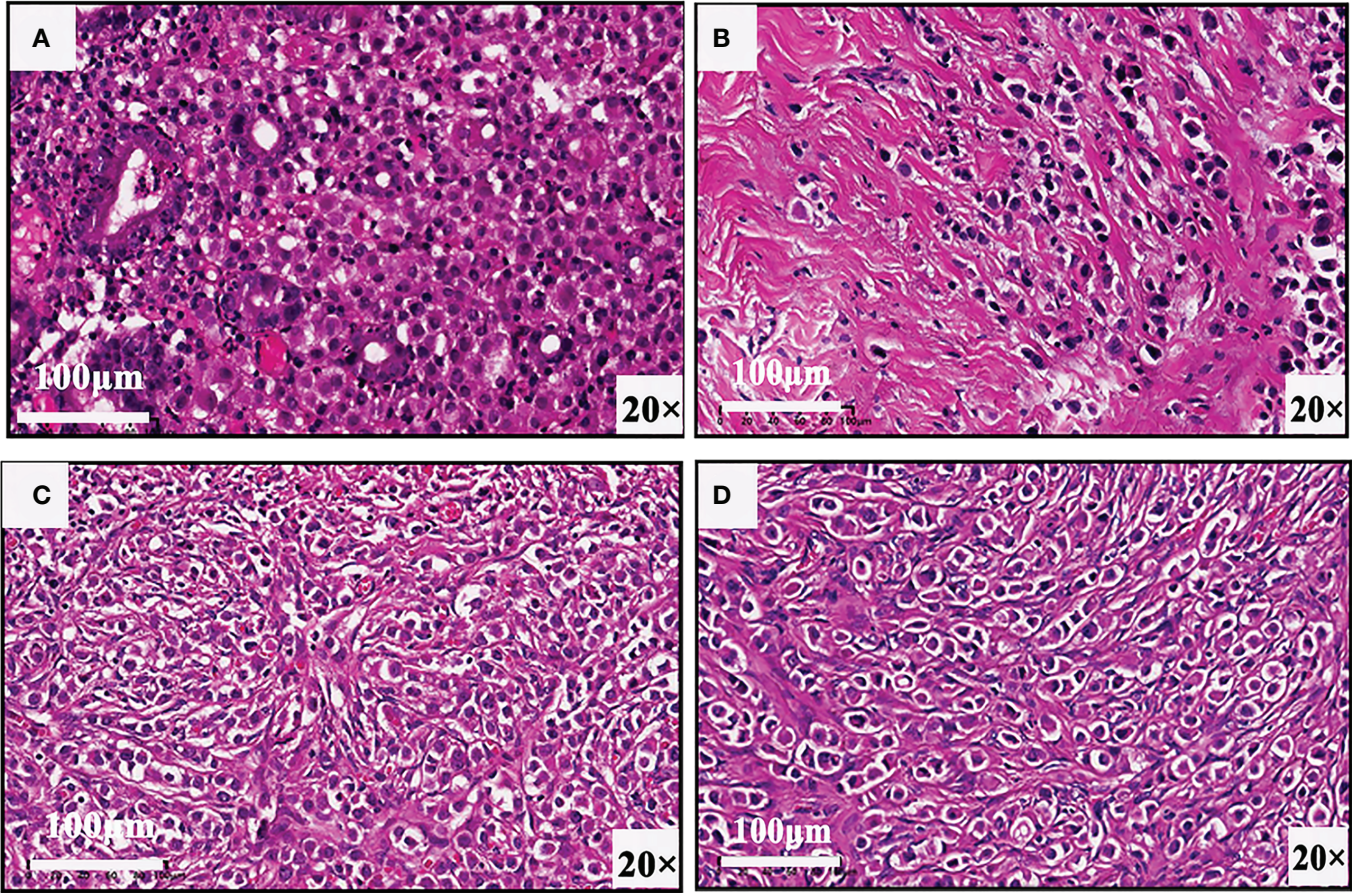
Biopsy results of **(A)** gastric wall, **(B)** pelvic nodule, **(C)** right ovary, and **(D)** left ovary confirm that there are gastric, peritoneal, and ovarian metastases from breast cancer.

In view of these auxiliary examination results, the patient underwent abdominopelvic exploration and pathological examination. In addition to gastric metastasis, histopathological and immunohistochemical examinations of bilateral adnexectomy and peritoneal nodule confirmed that both the peritoneum and bilateral ovaries had metastases from breast cancer (**Figure 3**). Subsequently, she received three consecutive days of intraperitoneal hyperthermic perfusion chemotherapy (paclitaxel 120 mg once a day). She is in a fair condition at present, and we will continue to follow her up in the future.

## Discussion

Gastrointestinal metastases can sometimes be seen in patients with cancers of the breast, kidney, lung, and malignant melanoma. Breast cancer is the second type of cancer (after lung cancer) that determines gastrointestinal metastasis (1). Notably, different histological types of primary breast cancer tend to have different metastasis sites. Compared with invasive ductal cancer, metastases from breast cancer to the gastrointestinal tract, peritoneum, ovaries, and uterus are more likely to occur in invasive lobular cancer (2, 3). However, this is a case of mixed breast cancer. When breast cancer metastasizes to the gastrointestinal tract, the onset is hidden, and there are often no special manifestations. Some patients with breast cancer will even get hospitalized with gastrointestinal discomfort as the first symptom (4). The tumor cells of gastric metastasis from breast cancer usually grow diffusely under the mucosa and muscularis, resulting in localized or diffuse gastric wall thickening, which is difficult to be distinguished from primary gastric malignancies (5, 6). Pathological examination and immunohistochemistry are gold standards for diagnosis. Additionally, research shows that HNF4A alone could be a marker for distinguishing primary gastric cancer from breast metastasis (7).

Ovarian metastasis is frequently encountered during the course of breast cancer, concerning one woman in five among those suffering from the disease. These secondary ovarian lesions are usually small and bilateral with a non-cystic pattern and are more likely to be from the primary infiltrating lobular carcinoma of the breast (8). Previous studies suggest that the size of the tumor, positive lymph nodes, and the histological type are all related to the occurrence of ovarian metastasis from breast cancer. Researchers also emphasize the importance of measuring the level of the breast marker CA153 and the ovarian marker CA125 in the serum (9). Similarly, pathological and immunohistochemical results are the gold standard for the diagnosis of ovarian metastasis of breast cancer. Bilateral, solid, small-sized, and highly vascularized masses are characteristic features of ovarian metastasis on macroscopic examination (10).

Peritoneal metastasis from breast cancer is a challenging clinical presentation. There is a lack of knowledge syntheses and specific recommendations for the management of breast cancer peritoneal metastases. The majority of pathology and imaging reports demonstrate that breast cancer peritoneal metastasis is mainly associated with invasive lobular carcinoma and the following intrinsic subtypes: HER2-enriched, luminal B, and basal-like (11, 12). Therefore, for patients with breast cancer, doctors should pay attention to identifying these metastatic cancers to develop correct treatment plans, and ^68^Ga-FAPI PET/CT will stand out in the process of auxiliary diagnosis.

Most patients with peritoneal metastasis have poor prognosis, with only a minority of the patients having a survival time of more than 6 months (11, 12). In addition, research shows that the median survival time until the gastric involvement was 19 months (range, 0–41 months) (13). At present, there are still few original studies on the prognosis of gastric, peritoneal, and ovarian metastases of breast cancer.

FAPI, a newly developed tumor imaging target, can specifically bind to FAP *in vivo*, which is overexpressed in cancer-associated fibroblasts in many primary solid tumors and metastases. Many recent studies have demonstrated that ^68^Ga-FAPI is superior to ^18^F-FDG in tumor detection in many types. Compared with ^18^F-FDG, ^68^Ga-FAPI shows several advantages, including a better tumor-to-background ratio, independence of blood glucose levels, rapid renal clearance, and feasibility of rapid image acquisition (14, 15). Many recent studies have demonstrated that ^68^Ga-FAPI is superior to ^18^F-FDG in the detection of many types of tumors, especially some malignant lesions of the gastrointestinal tract and peritoneum, with higher tracer uptake in most primary and metastatic lesions. One of the possible reasons is that ^18^F-FDG PET/CT imaging is often interfered by non-specific gastrointestinal physiological uptake in the abdomen and the pelvis, which may cause diagnostic bias, whereas the uptake of ^68^Ga-FAPI is not affected by this. Moreover, ^18^F-FDG may appear false negative in some malignant lesions with inactive glucose metabolism, such as gastrointestinal tumors with more signet ring cells and mucinous tissue in which ^68^Ga-FAPI will show great advantages (16–21). This case may be an example that can guide physicians in making better treatment strategies.

## Conclusion

Gastric and ovarian metastases of breast cancer can present with clinical and imaging characteristics resembling primary malignancy. Even pathological changes are difficult to identify, and immunohistochemistry is helpful for differential diagnosis. These similarities can cause significant diagnostic difficulties, with a subsequent therapeutic delay and a potential adverse outcome. However, how to accurately evaluate tumor staging and restaging before treatment is an important basis for clinical decision-making. Therefore, patients with a history of breast cancer should be considered for the possibility of developing distant metastasis if abnormal changes are found in the above parts of the body. It is worth noting that PET/CT can detect regional metastatic lymph nodes and distant metastasis through one-stop imaging, which is becoming more and more significant in clinical staging and restaging of tumors. In particular, for malignant tumors in the abdomen and the pelvis, such as gastrointestinal tumors and peritoneal carcinoma, whether primary or metastatic, ^68^Ga-FAPI may have a greater advantage over ^18^F-FDG, with a higher detection rate.

## Data availability statement

The original contributions presented in the study are included in the article/supplementary material. Further inquiries can be directed to the corresponding authors.

## Ethics statement

This study was approved by the institutional review committee of our hospital (No.2021069). The patients/participants provided their written informed consent to participate in this study.

## Author contributions

TL and XJ were involved in the drafting and final editing of the report. They contributed equally to this work and share first authorship. ZZ and XC processed the images, and JW followed up with the patient. XZ and JZ, the corresponding authors, designed the study. All authors contributed to the article and approved the submitted version.

## Funding

This work was financially supported by the Hebei Provincial Department of Science and Technology in China (No. 20377728D).

## Acknowledgments

Thanks are due to all authors for assistance with the case report and the valuable discussion. In addition, funding from the Hebei Provincial Department of Science and Technology in China is gratefully acknowledged.

## Conflict of interest

The authors declare that the research was conducted in the absence of any commercial or financial relationships that could be construed as a potential conflict of interest.

## Publisher’s note

All claims expressed in this article are solely those of the authors and do not necessarily represent those of their affiliated organizations, or those of the publisher, the editors and the reviewers. Any product that may be evaluated in this article, or claim that may be made by its manufacturer, is not guaranteed or endorsed by the publisher.
